# Editorial: Towards 2030: sustainable development goal 9: industry, innovation and infrastructure. A sociological perspective

**DOI:** 10.3389/fsoc.2024.1491091

**Published:** 2024-11-12

**Authors:** Andrzej Klimczuk, Grzegorz Piotr Gawron, Magdalena Klimczuk-Kochańska, Piotr Toczyski

**Affiliations:** ^1^Department of Social Policy, Collegium of Socio-Economics, SGH Warsaw School of Economics, Warsaw, Poland; ^2^Institute of Sociology, Faculty of Social Sciences, University of Silesia in Katowice, Katowice, Poland; ^3^Faculty of Management, University of Warsaw, Warsaw, Poland; ^4^Media and Communication Department, Institute of Philosophy and Sociology, The Maria Grzegorzewska University, Warsaw, Poland

**Keywords:** SDG9, environmental regulation, industrial policies, innovation, sustainable industrial development, technology transfer

## Overview

This Research Topic explores the ninth Sustainable Development Goal (SDG), which aims to build resilient infrastructure, promote inclusive and sustainable industrialization, and foster innovation, particularly in the context of post-COVID-19 pandemic recovery. The pandemic significantly impacted the manufacturing sector, leading to a global production drop, job losses, and disrupted supply chains, with less technology-intensive industries taking longer to regain ground. Despite these challenges, the United Nations highlights opportunities to enhance industrialization and technology distribution, emphasizing, among other things, the need to expand mobile broadband networks, increase research and development investment, and improve rural road connectivity.

The Research Topic “*Towards 2030: sustainable development goal 9: industry, innovation and infrastructure. A sociological perspective*” was edited in cooperation with two journals: “Frontiers in Sociology” and “Frontiers in Ecology and Evolution.” The presented Research Topic includes eight original research articles of prepared in total by 30 authors who deal with subjects covering issues such as green development, environmental regulation, carbon reduction, institutional development, digital economy, innovation, and technology transfer. The articles comprising this Research Topic are organized according to three themes.

## Theme I: selected challenges of the inclusive and sustainable industrialization

The team of Cao et al. analyzed Chinese firms that are internationalizing rapidly, challenging mainstream theories of corporate growth. Using data from Chinese industrial enterprises, the study examines the relationship between exports and innovation through a recombinatory framework integrating resource-based and institution-based views. Findings reveal a *U*-shaped relationship between exports and innovation, influenced by provincial institutional factors, with higher institutional development levels reversing this relationship, offering significant insights for managing export-driven innovation. Moreover, Zhang and Wang present a study that provides evidence for another *U*-shaped relationship in the industrial sector. The authors argue that environmental information disclosure is crucial for promoting carbon neutrality and sustainable development amidst economic growth and environmental degradation. Using data from Chinese A-share listed companies, their study finds a *U*-shaped relationship between environmental information disclosure and corporate sustainable growth, initially decreasing but then increasing, mediated by innovation inputs. The association of these factors is influenced by firm size and equity incentives, which are more pronounced in non-state enterprises than state-owned ones.

## Theme II: regulation and measures supporting innovation

The following section covers studies that further examine environment-related innovations in industrial development. Chen et al. examine the impact of environmental regulation on industrial green development in China, using data from 30 provinces between 2006 and 2018. Employing various empirical models, their research reveals that the environmental regulation index significantly promotes green development, with specific regulations influencing technological progress and fiscal decentralization. Peng and Zhang continue to discuss the regulatory conditions in studying industry-university-research cooperation (IURC). According to the authors, IURC is a strategic measure to boost the international competitiveness of China's high-tech manufacturing (HTM) sector, but its links to environmental efficiency (EE) are underexplored. The presented investigation uses advanced models to analyze the impact of IURC on HTM's EE, revealing that, while IURC has a significant negative direct effect, it positively influences EE indirectly through research and development investment. The findings underline the urgent need to improve EE in China's HTM industry, especially in central and western regions, by promoting IURC and increasing investment in environmental technology. Finally, Zhou and Peng present the results of the study regarding the promotion of technology transfer. The authors argue that this is a crucial strategy for enhancing industrial innovation in China. Yet, its impact on the green innovation efficiency (GIE) of the high-tech industry (HTI) remains under-researched. The article presents a three-stage network data envelopment analysis (NDEA) model and regression models to evaluate the effects of domestic technology acquisition (DTA) and foreign technology introduction (FTI) on GIE, finding that DTA significantly boosts GIE. At the same time, FTI has a positive but not statistically significant impact. These insights highlight the need for tailored technology transfer policies to improve green innovation across different provinces in China's HTI.

## Theme III: regional and local conditions for green development

The last part of this Research Topic is opened with the study by Liu et al., which focused on the integrated development of industries in China that increasingly focus on achieving carbon neutrality. Analyzing data from 30 Chinese provinces, this study reveals that collaborative agglomeration between productive service and manufacturing industries significantly improves regional green development efficiency, with technological innovation playing a key mediating role. Additionally, the research identifies a non-linear relationship and regional heterogeneity in the impact, leading to policy recommendations for enhancing industrial synergy, promoting technological innovation, and boosting regional green productivity. Ma et al. show another example related to challenges in green development. Integrating digital technology and China's national carbon neutrality strategy can reduce urban carbon emission intensity (CEI). Analysis of data from 110 cities in the Yangtze River Economic Belt (YREB) shows that the development of the digital economy lowers CEI by promoting industrial structure optimization and green technology innovation and exerts a positive spatial spillover effect on surrounding cities. The final chapter of this section by Shen et al. continues on these Research Topics. The authors argue that promoting digital technology is crucial for addressing global climate change and achieving carbon neutrality goals. An econometric analysis of Chinese cities from 2006 to 2020 indicates that digital technology significantly reduces carbon emission intensity and improves carbon emission efficiency through green technological innovation and reduced energy intensity. The study highlights the role of digital technology in accelerating knowledge transfer and creating spillover effects that aid in carbon emission reductions, thus supporting the green transformation of the economy and society.

## Conclusion

The research results contained in the articles in this Research Topic allow for the proposal of at least five directions for further research. These are (1) social and cultural aspects of innovation regulation and technology transfer (see UNCTAD, 2014; OECD, 2021); (2) multi-level, cross-sectoral, and multi-sectoral cooperation of various stakeholders in the development of sustainable industry, innovation, and infrastructure (see Arbeiter and Bučar, 2021); (3) regional and local bottom-up solutions in the fields of green development, their scalability, feedbacks from environmental change, degrowth, and community resilience (see Marradi and Mulder, 2022); (4) advances in the access of various industries to digital infrastructures, information, and communications technologies as well as artificial intelligence solutions (see Diodato et al., 2022; ECLAC, 2021); and (5) new ideas for support of SDGs in the fields of technological policy, industrial policy, and innovation policy such as the mission-oriented innovation and industry 5.0 concept (see UNCTAD, 2017; Dixson-Decleve et al., 2022).
